# The Non-Denatured Processing of *Brasenia schreberi* Mucilage—Characteristics of Hydrodynamic Properties and the Effect on In Vivo Functions

**DOI:** 10.3390/foods13121824

**Published:** 2024-06-11

**Authors:** Tingyang Ai, Jiawei Wan, Xiujuan Yu, Jiao Liu, Cong Yin, Lindong Yang, Hong Liu, Rui Qin

**Affiliations:** 1 Hubei Provincial Key Laboratory for Protection and Application of Special Plant Germplasm in Wuling Area of China, College of Life Sciences, South-Central MinZu University, Wuhan 430074, China; 2021204@mail.scuec.edu.cn (T.A.); 2020037@scuec.edu.cn (J.W.); yuxiujuan0531@163.com (X.Y.); jiao.liu@scuec.edu.cn (J.L.); yincong@scuec.edu.cn (C.Y.); liuhong@scuec.edu.cn (H.L.); 2 Conservation and Comprehensive Utilization Engineering Center of Biological Resources in Southern Minority Areas, College of Life Sciences, South-Central MinZu University, Wuhan 430074, China; lyndonyang153@foxmail.com

**Keywords:** *Brasenia schreberi* mucilage, freeze-drying, rheological property, microstructure, gut microbes, colitis

## Abstract

Food non-denatured processes, such as freeze-drying and grinding, are commonly applied to raw materials with good bioactive functions. Although the functional components are maintained, whether structural and physical changes impact the in vivo function is often ignored in practical situations. *Brasenia schreberi* mucilage (BSM) has a significant alleviation effect on DSS-induced colitis. This work focused on the influence of non-denatured manufacture on the colonic benefits of BSM-based products. First, three forms of products including fresh mucilage (FM), freeze-dried products (FS), and freeze-dried powder (FP) were prepared. Then, their in vitro physiochemical properties were compared, analyzing their influence on the gut inflammation degree, microbial composition, and SCFA production in mice. The results suggested that the water retention rate of FS and FP was decreased to 34.59 ± 3.85%, and 9.93 ± 1.76%. The viscosity of FM, FS, and FP was 20.14 Pa∙s, 4.92 Pa∙s, and 0.41 Pa∙s, respectively. The freeze-drying and grinding process also damaged the lamellar microstructure of BSM. Then, animal tests showed that colitis mice intervened with FM, FS, and FP had disease activity scores of 2.03, 3.95, and 4.62. Meanwhile, FM notably changed the gut microbial composition and significantly increased propionate and butyrate levels. It seemed that the distinct colitis alleviation efficacy of BSM-based products is attributed to different hydrodynamic properties in the gut. FM had relatively higher viscosity and correspondingly high nutritional density in the gut lumen, which stimulates Firmicutes growth and promotes butyrate production, and thereby exhibited the best efficiency on protecting from colitis.

## 1. Introduction

Food processing is an important part of the modern food industry. A range of processing technologies, including heating, freeze-drying, high pressure treatment, microwave processing, etc. are frequently induced to food systems to obtain better quality and more consumer preference-oriented attributes [1]. A lot of studies have investigated the influence of processing method on food consuming quality, such as taste, texture, flavor, and shelf-life, and in order to achieve the goal of fine-tuning those techniques [2]. However, for modern people, various of commercial products are preferred to be consumed due to their health benefits rather than the traditional role of energy supply or appetite satisfaction. So, it is important to study the bioactive functions of processed foods. For this reason, this paper studied the potential relationship among the processing methods, physiochemical properties, and physiological effects of food materials. As the global intake of processed foods increases, the health benefits of their physiochemical characteristics needs to be examined.

Among various kinds of food processing methods, lyophilization (with or without subsequent grinding) was one of the most used manufacturing processes, which has been preferred as a non-destructive method [3]. As low temperature avoids the denaturation of nutrients and the loss of bioactive molecules, the freeze-drying process would preserve the nutritional or biofunctional aspects of the raw material more than many other processing techniques [4]. However, the physiochemical characteristics alter during the process [5]. Thus, lyophilization is suitable to be applied to food material to investigate the potential functional effects of their physical properties.

*Brasenia schreberi* J.F. Gmel is a perennial freshwater macrophyte which belongs to the family Cabombaceae [6]. In Asia, BS has long been consumed as an aquatic vegetable. This plant is special because the undersides of young leaves are coated with a thick, clear mucilaginous layer, which brings a unique texture of BS. The main component of the mucilage (denoted as BSM in the paper) is polysaccharides [7]. Recently, studies have reported that this polysaccharide could modulate the lipid metabolism of high-fat diet fed hamsters by down-regulating the low-density lipoprotein and total cholesterol levels [8]. In vitro antioxidation assessment showed that the polysaccharide exhibited strong free radical scavenging activities for 2,2-diphenyl-1-picrylhydrazyl (DPPH and 2,2′-azinobis-(3-ethylbenzothiazoline-6-sulphonate)(ABTS+) radicals [9]. In our previous work, the mucilage alleviated ulcerative colitis in mice [10]. Ulcerative colitis is a chronic colorectal inflammatory disease. It commonly manifests as intestine mucosal damage, ulceration, and persistent diarrhea [11]. This disease has recently emerged as a public health issue, but the pathogenesis remains unclear. Drug therapy exerts good efficacy but has problems related to long treatment cycles and side effects. So, it is important to find alternative compounds such as food-derived active substances to prevent and treat the development of colitis. Recently, polysaccharides from natural sources have been reported to be beneficial to colonic health with advantages of good therapeutic effect, non-toxicity, safety, and good biocompatibility [12]. As a raw material, BSM could potentially be developed into functional products with anti-inflammatory effects in the treatment of ulcerative colitis. In our preliminary experiment, we found that the nutritional content (polysaccharides, polyphenols, and protein) of BSM was almost unchanged after freeze-drying, but the viscosity decreased. A recent study showed that foods with different flow characteristics had effects on gut microbial modulation [13]; however, whether the processing method changes its bioactive functions needs to be considered.

Herein, lyophilization or/and grinding processes were employed in this work to prepare three different forms of BSM-based products, which included fresh mucilage, its freeze-dried sheet-like products, and the freeze-dried grinding powder products. This work investigated the fine structure and rheological properties after lyophilization or lyophilized grinding. Then, using colitis mice as the target animal model, the efficacy of the three BSM-based products alleviating colitis was evaluated. Furthermore, by determining the gut microbial alteration andshort chain fatty acids (SCFAs) production, the different colonic health effects of BSM-based products resulting from different processing methods were discussed from the aspect of hydrodynamic contributions to gut physiology.

## 2. Materials and Methods

### 2.1. Materials and Reagent

BS was collected from a germplasm resources nursery (Lat. 108°47′ N, 30°11′ E) in Fobaoshan, Lichuan, Hubei Province, China, and preserved in acetic acid for storage. Dextran sodium sulfate (DSS) was obtained from MP (36–50 KDa, MP Biomedicals, Ontario, CA, USA). The other chemical reagents used in this study were all of analytical grade.

### 2.2. Sample Preparation

BS were gently washed with flowing deionized water and drained for two hours. Then, with a 5 s shearing by a blender machine (MX-SS1, Panasonic, Osaka, Japan) and a centrifugation process (Centrifuge 5810R, Eppendorf, Hamburg, Germany) at 4 °C, 10,000× *g* for 10 min, the fresh mucilage (FM) was separated from the BS plant. Next, the FM samples were pre-frozen at −20 °C for 2 h and then transferred into the freeze-drying machine. The vacuum degree was 10 Pa, and the duration time was 48 h. Followed by lyophilization, FM was freeze-dried as sheet-like samples, which was denoted as FS in this paper. Subsequently, conducted by a crusher machine, FS was further processed into power samples, which was denoted as FP in the following content.

### 2.3. Water-Holding Capacity

The determination of water-holding capacity was conducted by a centrifugation method [14]. First, the dried FS and FP samples were dispersed into water at a weight ratio of 0.2%, which was the previously determined solid content ratio of the FM sample. Then, 30 mL of FM and hydrated FS and FP was separately transferred into tubes and went through a centrifugation process at 4 °C, 3000 rpm for 5 min. The supernatant liquid was carefully removed by pipette. The water-holding ability was determined by the proportion of retained mass of each sample.

### 2.4. Rheological Tests

The rheological tests of FM, FS, and FP samples were performed on a DHR-2 rheometer (TA Instruments, New Castle, DE, USA) at room temperature (25 °C). Followed by the loading sample, the edge of the geometry was carefully cleaned. Before tests, each sample was equilibrated for 5 min. For the steady shear test, a cone and plate geometry (60 mm diameter) were used with a gap of 0.5 mm. The measurements were performed within a shear rate range from 0.1 to 100 s^−1^. For the strain and frequency sweep test, a parallel plate geometry (40 mm diameter, 0.5 mm gap) was used. Tests of dynamic strain sweep were conducted at 1 Hz to determine the linear viscoelastic regime with a strain range from 0.1 to 1000%, and the dynamic frequency sweep ranging from 0.1 to 10 rad s^−1^ was conducted under a constant strain of 10%.

### 2.5. SEM Analysis

The microstructures of FM, FS, and FP were analyzed using a Scanning Electronic Microscope (JSM-6390LV; JEOL Ltd.; Tokyo, Japan). The three samples were firstly placed in −80 °C for 2 h. After the samples were frozen, they were transferred into a freeze-drying machine. Experiencing a lyophilization process for 48 h, the dried samples were subjected to microstructure observation.

### 2.6. Cryo-SEM Tests

The microstructures of FM, FS, and FP were characterized by Cryo-SEM (8100, Hitachi Regulus, Tokyo, Japan) equipped with a Quorum PP 3010T cryogenic transportation device. Followed by rapid freezing with liquid nitrogen slush, the samples were transferred to a freezing chamber and fractured using a cryo-knife. Afterwards, testing samples went through stepwise sublimation at temperatures of −100 °C for 15 min. Then, gold sputtering was applied, and observations were conducted using a Hitachi SU8010 SEM (SU8010, Hitachi, Tokyo, Japan) with an Oxford EDS detector at −130 °C.

### 2.7. Animal Experiment

Fifty 8-month-old C57BL/6 male mice were purchased from the SPF (Beijing) Biotechnology Co., Ltd. (Beijing, China) and kept at the Experimental Animal Center of south–central Minzu University with a controlled environment (specified-pathogen-free, 12 h light/dark cycle, 22 ± 2 °C, 55 ± 5% humidity). Within the experiment, all treatments on rats were strictly in accordance with the National Research Council’s Guide for the Care and Use of Laboratory Animals. Protocols used in this research were approved by the Hubei Experimental Animal Testing Station (License number: SCXK20200019).

**Figure 1:**
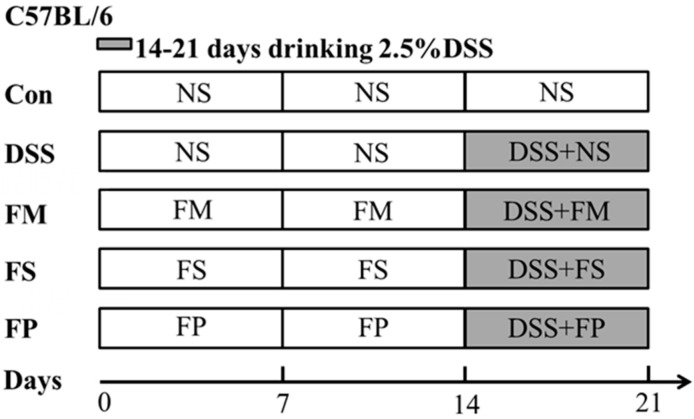
Schematic diagram of animal experimental design.

After one week of acclimatization, mice were randomly divided into five groups (*n* = 10), which were separately named as the Control, DSS, FM, FS, and FP group according to their diet intervention. The grouping information and respective treatment are depicted in Figure 1. Briefly, during the 3-week experimental period, the Control group and DSS model group were gavaged with saline. The three experimental groups were intervened with the FM, FS and FP samples with a dosage of 25 mg/kg body weight each day. In the third week, all mice were given an additional 2.5% DSS water except for the Control group. The food intake and body weight of each mouse were monitored daily.

### 2.8. Disease Activity Index (DAI) Evaluation

DAI was codetermined by the condition of body weight, diarrheal, and fecal bleeding [15]. Specifically, the DAI value was calculated by the sum of the scores of (i) body weight loss (no change, 0 score; 1–5% loss, 1 score; 5–10% loss, 2 scores; 10–15% loss, 3 scores; >15% loss, 4 scores); (ii) diarrhea (normal, 0 score; loose stools, 2 scores; watery diarrhea, 4 scores); and (iii) hematochezia (no bleeding, 0 score; slight bleeding, 2 scores; gross bleeding, 4 scores).

### 2.9. Colonic Histomorphology Analysis

After sacrifice and colon tissue collection, the colonic length was firstly measured for each mouse. Then, histopathological analysis was performed on distal colon tissue. Briefly, colon samples were fixed in 4% polyformaldehyde, embedded in paraffin, and then sliced into sections of 4 µm thickness. Followed by staining with hematoxylin and eosin, histological evaluation was conducted using a scoring system by two investigators [16]. Histology was scored as follows: epithelium: 0, normal morphology; 1, loss of goblet cells; 2, loss of goblet cells in large areas; 3, loss of crypts; 4, loss of crypts in large areas. Infiltration: 0, no infiltrate; 1, infiltrate around crypt basis; 2, infiltrate reaching to mucosa; 3, extensive infiltration reaching mucosa and thickening of the mucosa with abundant edema; 4, infiltration of the L. submucosa. The histological score was the sum of the two parameters.

### 2.10. Colonic Gene Expression

Total RNA was extracted from proximal colon tissue using a Universal RNA Extraction Mini Kit (Foshan Aowei Biological Technology Co., Ltd., Foshan, China). RNA quality was determined by an Implen NanoPhotometer-N80 spectrophotometer (Implen, München, Germany). The cDNA synthesis was carried out with MonScript™ RTIII Super Mix with dsDNase (two-step) (Mona Biotechnology Co., Ltd., Suzhou, China). Then, RT-qPCR was conducted with the MonAmp™ SYBR Green qPCR Mix kit using a Bio-Rad CT003142 real-time PCR system. The relative amount of transcript interleukin-1β (IL-1β) and tumor necrosis factor α (TNF-α) was normalized to the amount of TBP mRNA expression in the same cDNA. Data were analyzed according to the 2^−ΔΔCt^ method. Specific primers used for PCR amplification are exhibited in Table 1.

### 2.11. Fecal SCFAs Determination

The fecal SCFAs were determined based on the previous protocol [17]. Briefly, 100 mg feces was suspended with 500 µL of deionized water and centrifuged at 4 °C, 12,000× *g* for 10 min. Then, the supernatants were extracted by 25% metaphosphoric acid and 250 µL of diethyl ether. Afterwards, 25 µL of 2-ethylbutyric acid was added as an internal standard. The organic phase was further dehydrated using anhydrous sodium sulfate and went through 0.22 µm filters. Chromatographic analysis was performed on an Agilent 5977B GC system equipped with a flame ionization detector (FID) and GC-column (DB-WAX, 30 m × 250 µm × 0.25 µm). Nitrogen was applied as the carrier gas at a flow rate of 20.0 mL/min. The injected sample volume for GC analysis was 1 µL.

### 2.12. Microbial Analysis

Total DNA was extracted from a stool sample (about 200 mg). Primers 27F and 1492R were used to amplify the hypervariable V1–V9 regions of the 16S rDNA gene. The PCR reactions were carried out in 30 μL of reactions with 15 μL of Phusion^®^ High-Fidelity PCR Master Mix (New England Biolabs, Ipswich, MA, USA), 0.2 μM of forward and reverse primers, and 10 ng of templated DNA. PCR analyses were conducted using the following thermocycling program: 1 min of denaturation at 98 °C, 30 cycles of 10 s of denaturation at 98 °C, 30 s of annealing at 50 °C, 30 s of elongation at 72 °C, and a final extension step at 72 °C for 5 min. Then, the resulting PCR products were identified by 2% agarose gel electrophoresis and further quantified using QuantiFluor-ST (Promega, Madison, WI, USA).For microbial analysis, the raw fastq files were filtered using Cutadapt (V1.9.1). Then, all effective reads were clustered into operational taxonomic units (OTUs) using UPARSE Software (Uparse v7.1) with a similarity threshold of 98%. And the representative sequence of each OUT was aligned against the Silva Release138.1 database for taxonomy analysis. Biomarker strains with significant differences between comparison groups were identified at α = 0.05 (Kruskal–Wallis and Wilcoxon tests) using linear discriminant analysis (LDA). Species with an LDA score higher than 3.0 are shown by evolutionary cladistics.

### 2.13. Statistical Analysis and Visualization

The results were evaluated by one-way analysis of variance (ANOVA) with a subsequent mean comparison by Student’s *t*-test. Data were finally determined as mean ± SEM (*n* = 10) with at least three independent experiments. A *p*-value less than 0.05 was considered significant. The calculation and visualization process were performed using GraphPad Prism (v8.0.2, San Diego, CA, USA).

## 3. Results

### 3.1. Physiochemical Characteristics of FM, FS, and FP

#### 3.1.1. Water-Holding Capacity

**Figure 2:**
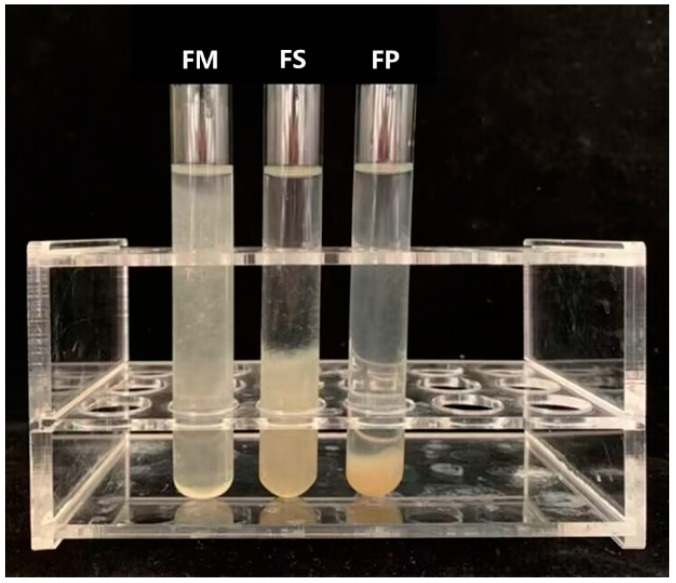
The appearance of FM, FS, and FP.

**Figure 3:**
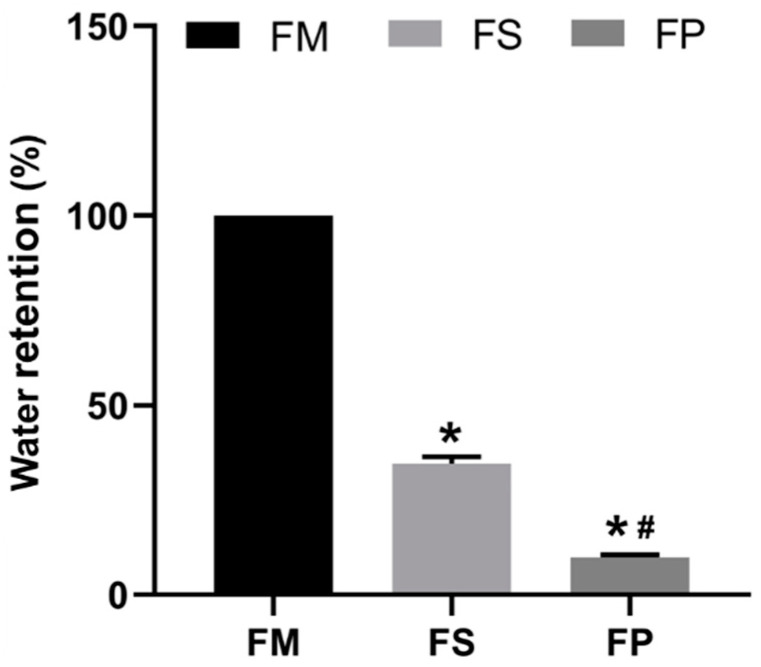
Water retention rate of FM, FS, and FP. * means significant difference compared to FM, # means significant difference compared to FS, *p* < 0.05.

The appearance of FM, FS, and FP is shown in Figure 2. The water-holding capacity was characterized by the water retention rate after moderate centrifugation. The results are shown in Figure 3. As the water retention rate of FM was denoted as 1, the results of FS and FP were 34.59 ± 3.85%, and 9.93 ± 1.76%, respectively. Apparently, the water retention rate FS was significantly lower than that of FM, and meanwhile, it was significantly higher than that of FP. The distinct water-holding rate of the three samples suggested that both freeze-drying and grinding would significantly damage the water-holding capacity of BSM.

#### 3.1.2. Rheological Properties

**Figure 4:**
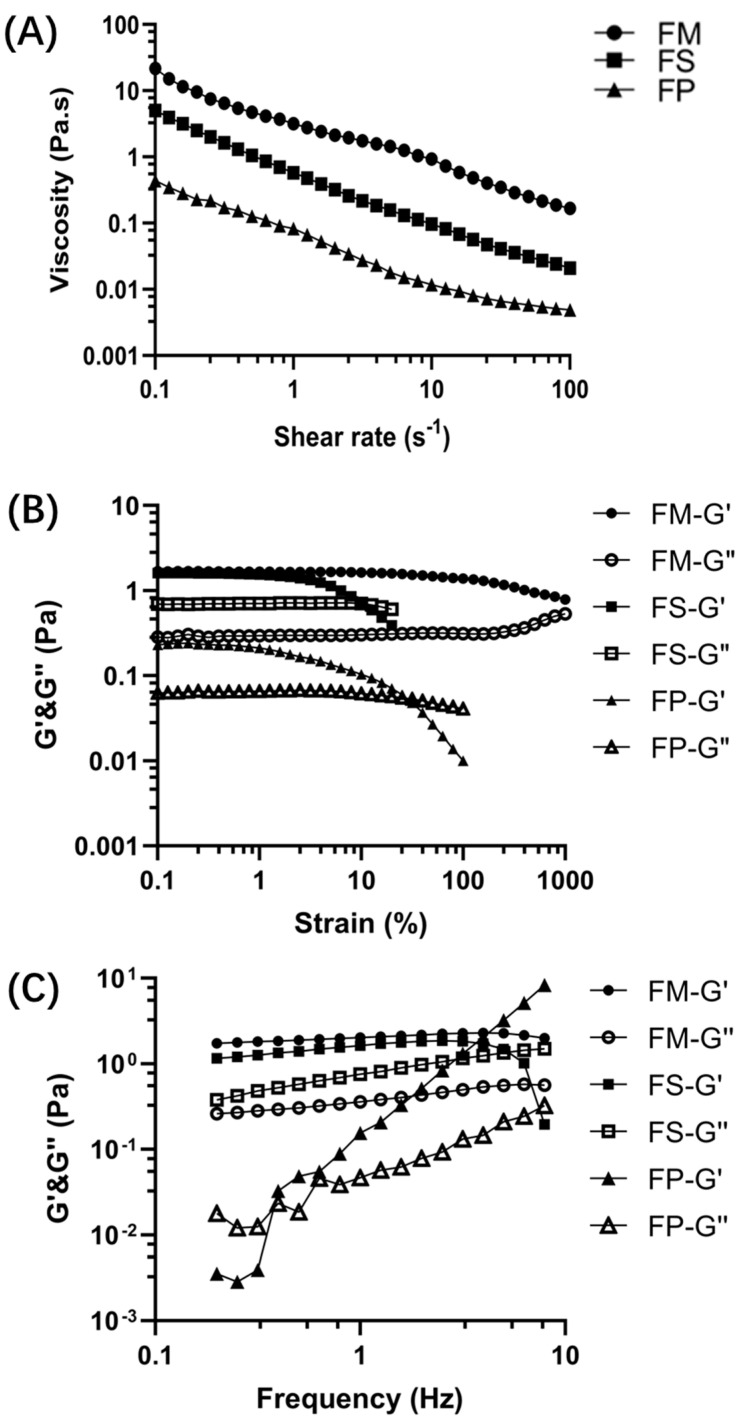
Rheological characteristics of FM, FS, and FP. (**A**) The steady flow behavior; (**B**) the strain sweep curves; (**C**) the frequency sweep curves.

The steady flow behavior of FM, FS, and FP is exhibited in Figure 4A. Generally, the viscosity of the three samples showed a linear-like descent as the shear rate increased from 0.1 to 100 s^−1^, which suggested a typical shear-thinning behavior. And the downward trend of the three curves was similar. When the shear rate began at 0.1 s^−1^, the viscosity of FM, FS, and FP was 20.14 Pa∙s, 4.92 Pa∙s, and 0.41 Pa∙s, respectively. Then, at any shear rate, the viscosity value of FM was higher than that of FS, and FS was higher than that of FP.

The results of strain sweep tests are presented in Figure 4B. First, FM and FS exhibited a typical linear viscoelastic region (LVR), during which both the storage moduli G′ and loss moduli G″ initially remained constant, and G′ was higher than G″. The LVR of FM was obviously larger than that of FS. Followed by LVR, the G′ of FM decreased and was finally met with upward G″. But for FS, G′ went downward and G″ was maintained. The situation of FP was different. Typical LVR was not observed. And the G′ of FP was initially higher than that of G″. With a constant decrease, G′ was finally intersected with G″ and became lower than G″. In addition, the initial G′ value of FM and FS was located at the same level, and it was notably higher than that of FP.

Then, the dependences of G′ and G″ on frequency were employed to characterize the viscoelastic behaviors of the three samples. The results are shown Figure 4C. For FM, both G′ and G″ were maintained a steady level. And G′ was higher than G″. In terms of FS, G′ held steady but ended with a drop; at the same time, G″ was slightly increased and finally intersected with G′. As for FP, both G′ and G″ exhibited a notable upward trend with increasing frequency, and the climbing trend of G′ was sharper than that of G″. Although G′ was lower than G″ at first, it exceeded G″ soon afterward.

#### 3.1.3. Microstructure

**Figure 5:**
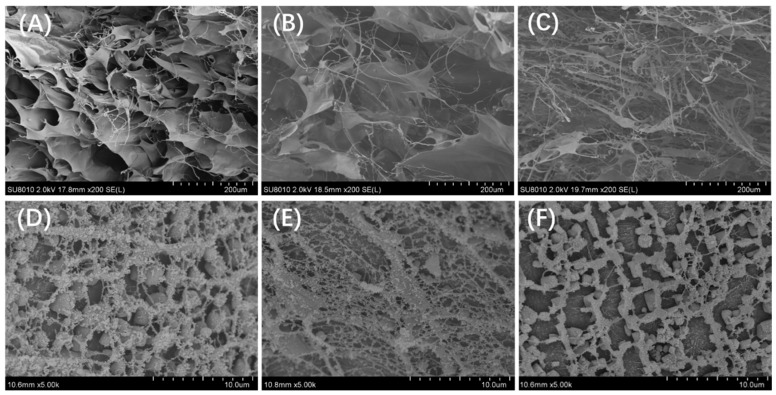
Scanning electron microscope image of FM (**A**), FS (**B**), and FP (**C**). Cryo-SEM images of FM (**D**), FS (**E**), and FP (**F**) samples. SEM images were captured at a magnification of ×200, and Cryo-SEM images were obtained at magnification of ×5k.

The microstructural information of FM, FS, and FP collected by SEM was presented in Figure 5A–C. Apparently, FM exhibited a three-dimensional cavity and a lamellar structure. When it came to FS, the lamellar structure was much smaller than that of FM, and there were some fibrous structures interspersed in the lamellar structure. However, for FP, the lamellar structure was no longer observed. It mainly contained fibrous structure, which were woven together. Then, the Cryo-SEM images showed more details about the micromorphology. The rapid freezing process involved in this technique minimizes ice crystal formation, maintains the spatial distribution of aqueous system, and thus prevents sample deformation under temperature and pressure plummet [18]. As shown in Figure 5D,E, all samples generally exhibited an uneven net-like structure with particle-like and fiber-like components within it. The structure of the FM and FS samples seemed dendritic, as the connecting fiber contained few trunks and a lot of branches. The structure of FM seemed more dimensional; by contrast, the network of FS was more plane. In terms of FS, it was much looser as the connecting fiber-like structures were notably decreased, and the dendritic features could hardly be recognized.

### 3.2. In Vivo Effect of FM, FS, and FP on Colitis Symptoms

#### 3.2.1. DAI Score

**Figure 6:**
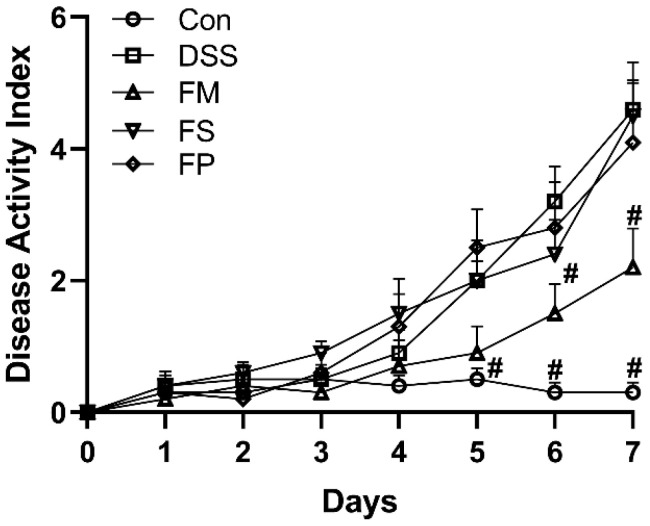
DAI scores for each group. Notes: # means significant difference compared to the DSS group, *p* < 0.05.

The colitis of each group was visualized by DAI scores; the higher the score, the more severe the symptoms. As shown in Figure 6, the score of the DSS group was significantly higher than that of the Control group, suggesting the success of experimental model establishment. Then, compared with the DSS group, the FS and FP groups exhibited significant difference, but the DAI of the FM group significantly decreased. It indicated that among the three samples, only FM had the alleviating effect of FM on colitis.

#### 3.2.2. Colonic Histopathological Evaluation

**Figure 7:**
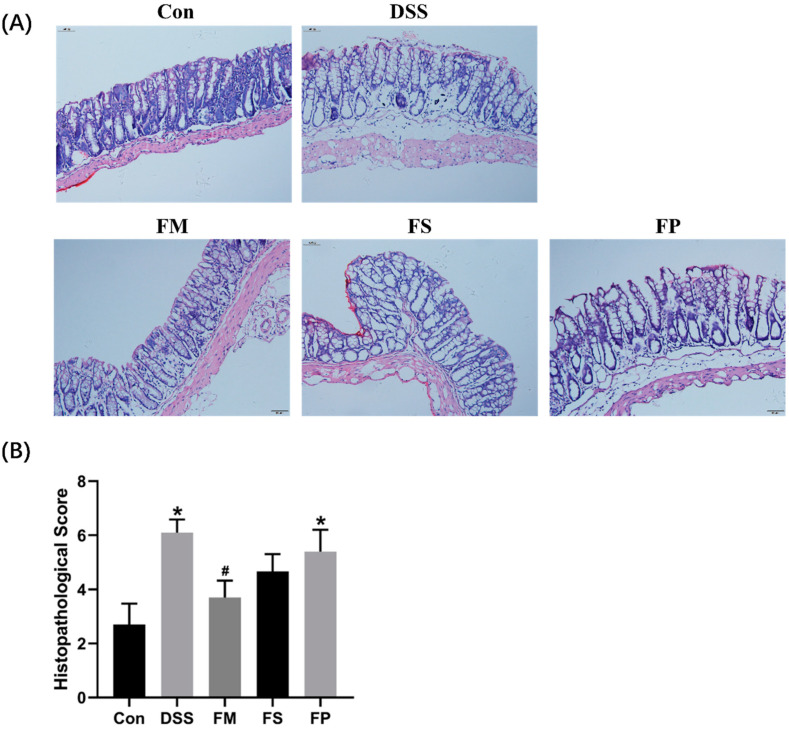
Colonic histopathological evaluation. (**A**) Colonic H&E staining images with a magnification of ×200. (**B**) Histopathological scores of colons. Notes: * represents significant difference compared to the Control group, # means significant difference compared to group DSS, *p* < 0.05.

As shown in Figure 7A, the histopathological analysis of colitis was conducted based on the evaluation of H&E staining pathology sections of colon tissue. For the mice of the Control group, the mucosal layer was apparently continuous and intact and had well-organized intestinal villi. For the DSS group, however, the pathological image was notably different. The arrangement of intestinal epithelial cells became disordered. The villi structure underwent atrophy, and the infiltration of inflammatory cells occurred, even in the submucosal layer. Those signs suggested typical ulceration and severe pathological damage of the colon tissue. As for the experimental FP and FS groups, villi morphology was greatly restored, but the inflammatory infiltration was still observed. By comparison, the FM group not only exhibited normal villi structure but also prevented cell infiltration. Based on these images, the colon pathological damage of each group was quantified and summarized in Figure 7B. The DSS group obtained the highest score, which was significantly higher than the Control group. And the FM group scored significantly lower than the DSS group. The FS and FP group were also lower than the DSS group, but the differences did not reach a significant level.

#### 3.2.3. Colonic Expression of Inflammatory Marker Genes

**Figure 8:**
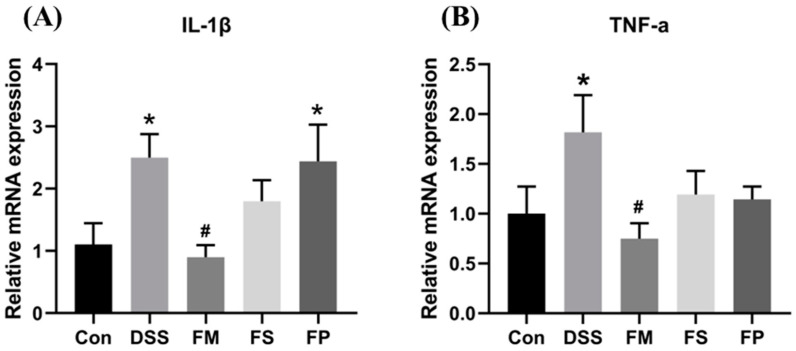
Gene expression level of cytokine IL-1β (**A**) and TNF-α (**B**) in proximal colon tissue. Notes: * represents significant difference compared to control, # represents significant difference compared to DSS, *p* < 0.05.

To confirm the colonic inflammation level of each group, the expression of cytokine IL-1β and TNF-α in the proximal colon was determined at the mRNA level. As shown in Figure 8, the relative mRNA expressions of IL-1β and TNF-α were significantly elevated in the DSS group compared to the Control group. However, those elevated expressions were remarkably down-regulated in the FM group. Although treatment of FS and FP decreased two cytokine levels, the differences did not reach a significant level.

#### 3.2.4. Gut Microbiota

**Figure 9:**
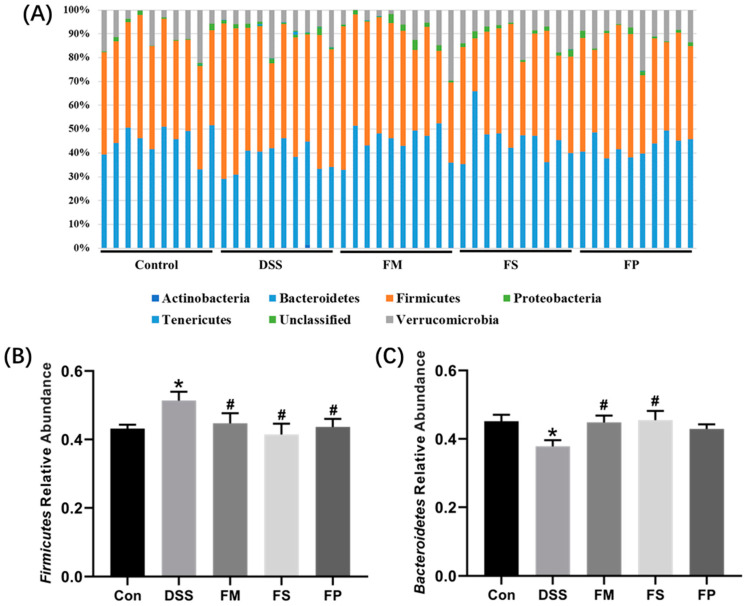
Gut microbiota composition at phylum level. (**A**) Relative abundance of the top six phyla of each mouse. (**B**) Relative abundance of Firmicutes for each group. (**C**) Relative abundance of Bacteroidetes for each group. Note: * means significant difference compared to control, # means significant difference compared to DSS, *p* < 0.05.

The effect of different BSM-based products on the gut microbial structure was investigated at the phyla level. As shown in Figure 9A, the microbial composition of each group was depicted by the most abundant phyla. Apparently, Firmicutes and Bacteroidetes were the two most abundant phyla. For Firmicutes, as shown in Figure 9B, it was significantly higher in the DSS group than in the Control group. The three experimental groups were all significantly lower than the DSS group. Then, for phyla Bacteroidetes, as depicted in Figure 9C, the DSS group was significantly lower than the Control group. The FM and FS groups were significantly higher than the DSS group; meanwhile, the FP group was within the same level as the DSS group.

**Figure 10:**
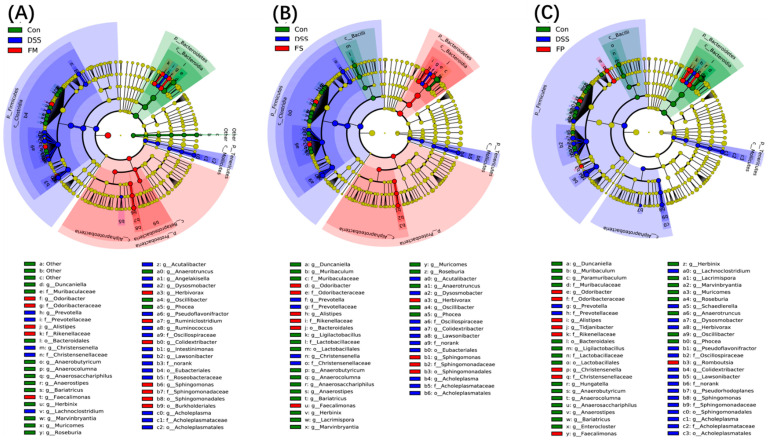
Taxonomic cladogram of LEfSe analysis among different diet groups. (**A**) Comparison among group Control, group DSS, and group FM; (**B**) Comparison among group Control, group DSS, and group FS; (**C**) Comparison among group Control, group DSS, and group FP. Note: Taxonomic levels represented by rings with phyla at the innermost ring and species at the outermost ring. Each circle is a member at that level, and each color represented the discriminating bacteria enriched in corresponding groups. The higher the relative abundance, the larger the circle.

To further discover the different modulations of the three BSM-based products on microbes, the gut microbiota of the three experimental groups were compared with that of the Control group and the DSS model group, respectively, using LEfSe analysis. The results are presented in Figure 10. Generally, by comparing the proportion of the red color among the three cladogram, the FP group had the minimum red area, suggesting there was a slight alteration in the FP group when compared with the DSS group. Figure 10A shows that Odoribacter, Alistipes, Feacalimonas, Herbivorax, Ruminiclostridium, Colidextribacter, and Sphingomonas were significantly enriched in the FM group, while those of the FS group were similar with to those of the FM group. But for the FP group, only Odoribacter, Alistipes, Feacalimonas, and Romboustia were significantly changed.

#### 3.2.5. SCFAs Production

**Figure 11:**
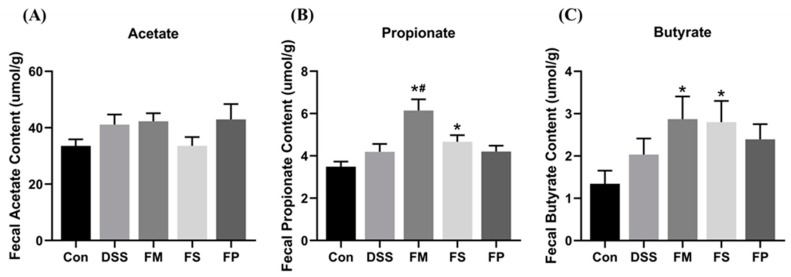
Fecal SCFA levels of acetate (**A**), propionate (**B**), and butyrate (**C**). Notes: * means significant difference compared to control, # means significant difference compared to DSS, *p* < 0.05.

Fecal SCFAs including acetate, propionate, and butyrate were determined for each group. The results are shown in Figure 11. For the level of acetic acid, there was no significant change among the groups. The contents of propionic acid and butyric acid in the FM group and FS group were significantly increased compared with the Control group. And the contents of propionic acid and butyric acid in the FM group were also significantly increased compared with the DSS group. By contrast, propionic acid and butyric acid in the FP group were not significantly changed compared with the DSS group.

## 4. Discussion

In this study, edible mucilage was separated from the BS plant and processed into three kinds of products by freeze-drying or/and grinding. After intervening the diet of DSS-induced colitis mice, the efficacy of three BSM-based products (FM, FS, and FP) protecting from colitis was evaluated. Importantly, by characterizing the in vitro rheological behavior, microstructural property, in vivo microbial alteration and gut SCFA productions, we investigated how the food processing method influences the product’s biological functions.

The basic information of BSM had been previously determined by our lab [10]. The moisture content was 98.72 ± 0.02%. In the solid portion, the polysaccharide content was 619.49 ± 70.79 mg/g. The polyphenols and protein content were 14.85 ± 20 mg PE/g and 18.03 mg/g, respectively. Accordingly, polysaccharides were the dominant portion of the solid substance of BSM, which was in accordance with the data reported in the literature [7].

With different processing methods, the microstructures of the three BSM-based products were remarkably different. FM exhibited a notable lamellar structure, which was in line with previous studies [19,20]. To be specific, the microstructure of the mucilage was characterized by a lot of nanosheets. These sheet-like structures in fact were the special network of cross-linked polysaccharides. Moreover, due to the abundant hydroxyl groups in polysaccharide molecules, plenty of hydrogen bonds existed within the spaces between the neighboring nanosheets, which made the mucilage retain substantial water molecules. That explained why FM had excellent water-holding capacity. Freeze-drying is a manufacturing process that commonly alters the spatial network of macromolecules by inducing various stresses to the food system, such as cold denaturation, freeze concentration, and ice crystal formation [21]. In this work, by affecting the spatial morphology of polysaccharide chains, lyophilization caused damage to the lamellar structure and brought about the collapse of interlayer spaces. These changes resulted in alterations in the structural property and a decrease in the water-retaining ability of products. Then, the grinding process certainly crushed down the lamellar structures in the mucilage. Thus, after lyophilization and the grinding process, as the lamellar structure broke down, the water-holding capacity of the FS and FP products remarkably degraded.

When different processing methods were used, the three BSM-based products exhibited different rheological behaviors. In the steady-shearing test, FM, FS, and FP all exhibited non-Newtonian shear-thinning behavior along the testing range. Shear-thinning commonly appeared in the disperse system that was dominated by high molecular weight materials. Because as the shear rate increased, the long chain of polymers was randomly positioned and became increasingly aligned in the direction of flow; hence, interactions or entanglement between the adjacent polymer chains led to lower frequencies [22]. Many plant-derived polysaccharides exhibited shear-thinning [23]. In this research, the existence of polysaccharide molecules was also responsible for the shear-thinning phenomenon. Moreover, the sheet-like polysaccharide network of mucilage made it more likely to yield to external pressure than a uniform three-dimensional network. So, the viscosity of FM was lower than that of many plant polysaccharides [23] and exhibited a downward trend in an initial shear range. Additionally, due to the high compliance of the lamellar network upon external stress, BSM had the potential to be developed as a biological super-lubricity material [24]. With lyophilization and the grinding process, the network structure of polysaccharides was damaged, leading to the decreased viscosity of FS and FP.

Afterward, evidenced by DAI evaluation, the colonic histopathological section, and the inflammation cytokine level in animal tests, three forms of BSM-based products surprisingly exhibited different alleviation effects on colitis. FM showed the best efficacy, and meanwhile, FP was marginally effective. It suggested that freeze-drying and grinding processes indeed influenced the in vivo functions of BSM. Extensive studies demonstrated that diet modulation on gut microbes played important roles in decreasing colonic inflammation and preventing colitis [25]. In this work, among the enriched genus in the FM group, Alistipes and Odoribacter had previously been reported to produce acetic acid and propionic acid [26] and thereby exhibit anti-inflammation effects in colites [27,28]. The significantly higher levels of SCFAs observed in the FM group supported this point. SCFAs have been well demonstrated to benefit colonic health by promoting barrier integrity, modulating immune response, and relieving inflammation activities [29]. At the same time, FP marginally changed the microbial composition and did not lead to significant SCFA production. Hence, our work again implied that colitis alleviation was closely associated with the gut environment. So, how different forms of BSM-based products resulted in different gut microbial compositions and SCFAs levels seemed to be important and needs to be discussed next.

The bioactive functions of food are often attributed to their specific bioactive constituents. However, our study found that in addition to what the bioactive component was, how the food was processed also greatly influenced the in vivo functions. In our work, the nutritional or bioactive components of the FM, FS, and FP groups were the same. But different processing methods gave them different physiochemical properties. First, FM, FS, and FP had distinct viscosity levels; FP had the lowest viscosity and FM had the highest. Hence, the three products probably exhibited distinct fluid characteristics upon their entrance into the GI tract. Under similar luminal conditions (including length, diameter, and peristalsis), lower viscosity corresponded to higher flow velocity in the gut, which was approximated as a one-dimensional tube [30]. Studies showed that small variations in fluid flow could lead to a large shift in bacterial composition [13,31]. With a low density of nutrient inflow, low amounts of SCFAs can be produced by fermentative growth, resulting in a moderate drop in luminal pH [13], which would allow Bacteroidetes to grow faster than Firmicutes, leading to a higher relative abundance of Bacteroidetes [32]. When nutrient density is relatively high, more fermentation takes place, and the elevated SCFA secretion leads to a stronger drop in pH, which leads to a growth advantage of Firmicutes [32]. Then, due to the change in phyla composition, the composition of SCFAs changed. At low nutrient inflow rates, where Bacteroidetes dominate, most host calories are derived from acetate and succinate, which are mainly secreted by Bacteroidetes [33]. In contrast, at high nutrient inflow where Firmicutes dominate, acetate and butyrate (mainly secreted by Firmicutes) are dominant [34]. In our study, within a certain time, lower flow velocity was correlated with higher nutritional density in a certain length of the gut luminal. So, FM had the highest level of Firmicutes, and it significantly promoted acetate and butyrate. The SCFA composition has important consequences for the host because different SCFAs are used differently and exert different physiological effects. Butyrate has been suggested to have significant health benefits and anti-inflammatory effects in the colon [35]. FM led to the highest level of butyrate and hence exhibited the best efficacy on colitis.

In addition to gut microbiota and metabolites, the effect of exogenous polymers on mucus rheology also tended to affect gut physiological function. Mucus is a critical barrier lining the walls of the colon, it serves as the nexus of host–microbe interactions, and it protects against microbial infiltration and physical insults [36]. Regarding the microstructure, the mucus layer in fact is a network, which is composed primarily of high-molecular-weight glycoproteins known as mucins and is held together by physical entanglements, chemical crosslinks, and electrostatic interactions [37]. Exogenous molecules might disturb those intermolecular forces and consequently disrupt the protective function of the mucus. For example, studies had demonstrated that low-molecular-weight fragments were able to compete as mucin-binding sites, inhibiting the mucin–mucin interactions, reducing intermolecular cross-link density and long-range order, and thereby weakening the mucus gel [38]. For some food polyelectrolytes, like carboxymethyl cellulose, a component commonly found in processed foods and associated with inflammation and obesity was reported to compress the colonic mucus hydrogel in mice and hence altered the structure of colonic mucus [39]. In our work, FP was the most structurally fragmented and would be more likely to disturb the mucin network. Moreover, as polysaccharides in BSM had been reported as acid polysaccharides [40], they could be negatively charged in the gut environment and had an electrostatic repulsion on mucin molecules. Hence, FP seemed to more easily to disturb the mucin network and thereby impact the barrier functions.

## 5. Conclusions

In summary, this study prepared three kinds of BSM-based products by processing via freeze-drying or/and grinding and then investigated how the food processing method influenced the product’s biological functions. We have shown that the non-denatured processes changed the in vitro rheological behavior and damaged the lamellar microstructure of BSM. Moreover, the alleviating effect on colitis was negatively impacted. The results suggested that the hydrodynamic properties of products could shape the microbial composition and SCFA production in the gut. FM had relatively higher viscosity, and correspondingly high nutritional density in the gut lumen, which stimulates Firmicutes growth and promotes butyrate production, and thereby, it exhibited notable efficiency on protecting from colitis. FS was structurally fragmented and would be more likely to disturb the mucin network through electrostatic force and then impact the function of the mucus barrier layer. Our work provides guidance for the large-scale industrial production of BSM-based foods to obtain the active functions at maximum extent. This work is preliminary but still provides direction for practical applications. Future studies warrant precise mechanistic research focusing on the effect of hydrodynamic properties on gut physiological response and will look at the diet strategy to obtain higher health benefits of specific functions.

## Figures and Tables

**Table 1:** Abbreviations and sequence of real-time PCR primers.

Gene Name	Abbreviation	Primer Sequence
TATA box binding protein	TBP	F 5′-CTACCGTGAATCTTGGCTGTAAAC-3′
		R 5′-AATCAACGCAGTTGTCCGTGGC-3′
interleukin 1β	IL-1β	F 5′-TGCCACCTTTTGACAGTGATG-3′
		R 5′-TGATGTGCTGCTGCGAGATT-3′
tumor necrosis factor-alpha	TNF-α	F 5′- CGATGGGTTGTACCTTGTCT-3′
		R 5′- GTACTTGGGCAGATTGACCT-3′

## Data Availability

The original contributions presented in the study are included in the article, further inquiries can be directed to the corresponding author.
